# Superior Mesenteric Artery Syndrome in Down Syndrome: A Case Report

**DOI:** 10.7759/cureus.31854

**Published:** 2022-11-24

**Authors:** Fuad Alshaibani

**Affiliations:** 1 Radiology, Salmaniya Medical Complex, Manama, BHR

**Keywords:** case report, computed tomography, nausea and vomiting, superior mesenteric artery syndrome, small-bowel obstruction, down syndrome

## Abstract

Down syndrome is a common chromosomal abnormality, which is associated with a wide spectrum of multiorgan complications, including gastrointestinal disorders. Superior mesenteric artery syndrome is a rare type of small intestinal obstruction caused by compression of the duodenum between the superior mesenteric artery anteriorly and the abdominal aorta posteriorly. A 12-year-old boy with Down syndrome came to the emergency department with a first seizure secondary to electrolyte disturbances. He had been having recurrent episodes of bilious vomiting. Computed tomography of the abdomen revealed a markedly distended stomach and proximal duodenum with a narrow aortomesenteric angle consistent with superior mesenteric artery syndrome. The patient received successful conservative treatment with aggressive fluid resuscitation, bowel decompression, and electrolyte correction. Child protection services found that the patient had rapid weight loss recently after parents were confronted with the confirmed diagnosis of Down syndrome in their child. Physicians need to consider superior mesenteric artery syndrome in any child with an intellectual disability presenting with a clinical picture of intestinal obstruction.

## Introduction

Superior mesenteric artery syndrome is a rare type of small intestinal obstruction caused by compression of the duodenum between the superior mesenteric artery anteriorly and the abdominal aorta posteriorly [1]. It may present acutely with a clinical picture of proximal intestinal obstruction with severe nausea and bilious vomiting. Patients with intellectual disabilities were found to be more prone to develop superior mesenteric artery syndrome [1]. In general, patients with Down syndrome have a relatively high prevalence of acute and chronic gastrointestinal disorders [2]. Here, I present the case of a young child with Down syndrome who had a seizure secondary to electrolyte disturbances related to recurrent vomiting due to superior mesenteric artery syndrome. Certain conditions have been associated with an increased risk of developing superior mesenteric artery syndrome which includes abdominal surgeries and eating disorders, which can cause significant weight loss that leads to a decrease in the amount of the mesenteric fat pad [3].

## Case presentation

A 12-year-old boy with Down syndrome was brought to our emergency department with a complaint of jerky movements involving his upper and lower extremities associated with frothy secretions from the mouth and upward rolling of the eyes. This episode lasted for 2 minutes and had a spontaneous resolution. The child appeared tired after the episode and fell asleep. He had no previous similar episodes. The mother reported that the child had been having recurrent episodes of vomiting for the last five days. The vomiting occurs with every meal and was large in amount. She described the vomitus as coffee-ground in appearance. The mother reported that the child did not have bowel movements for one week. The child was seen by a general practitioner one week ago and was diagnosed as having acute gastroenteritis and advised increased fluid intake. There was no history of fever, symptoms of the upper respiratory tract, or decreased appetite. The past medical history was non-contributory. The patient had no previous surgeries. He was up-to-date on the vaccination schedule. The review of the medical records revealed that the diagnosis of Down syndrome was conveyed to the family after birth. However, the parents were in denial and did not want to accept the diagnosis. On their last visit to the pediatric clinic, which was two weeks before the patient’s current visit, the karyotype results were discussed with the parents.

On physical examination, the child appeared to be drowsy. There were no signs of respiratory distress. The patient was dehydrated with dry mucous membranes. Vital signs revealed a normal temperature of 37.0°C, tachycardia with a pulse rate of 120 beats per minute, a normal respiratory rate of 15 breaths per minute, and a normal blood pressure of 105/68 mmHg. The capillary refill time was prolonged for more than 2 seconds. The abdominal examination revealed a generalized distention with mild tenderness. However, no organomegaly was noted. The cardiac examination revealed normal heart sounds with no murmurs.

Initial laboratory investigations revealed a hemoglobin level of 9.0 g/dL, a white blood cell count of 9,600 cells/μL, and a platelet count of 314,000/μL. Electrolyte levels were disturbed, including hyponatremia (127 mEq/L), potassium level of 3.3 mEq/L, low chloride level (70 mEq/L), and elevated bicarbonate level (43 mEq/L). The patient was admitted for further evaluation and management.

**Figure 1:**
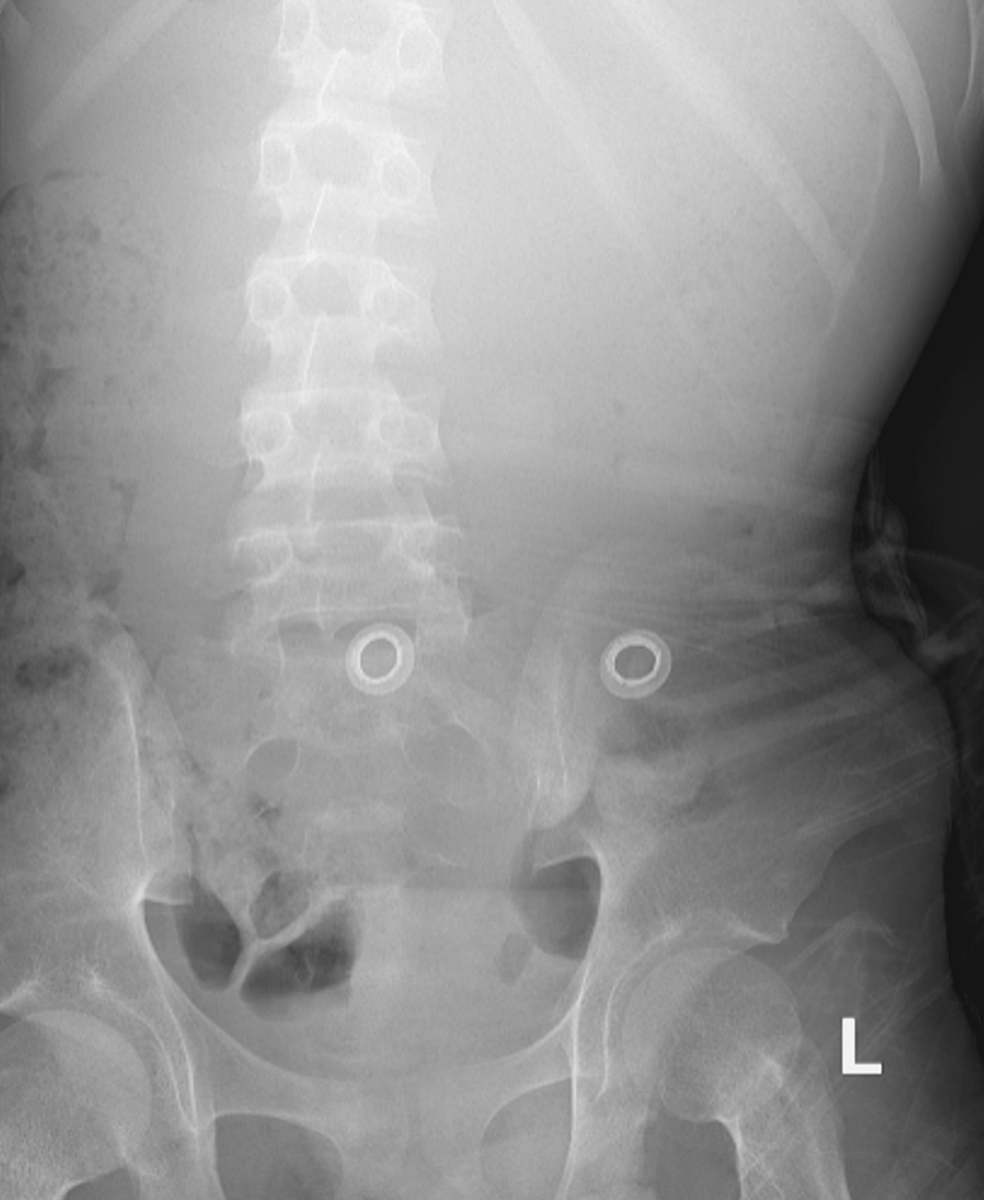
Plain abdominal radiograph shows markedly distended stomach with no small or large bowel dilatation.

**Figure 2:**
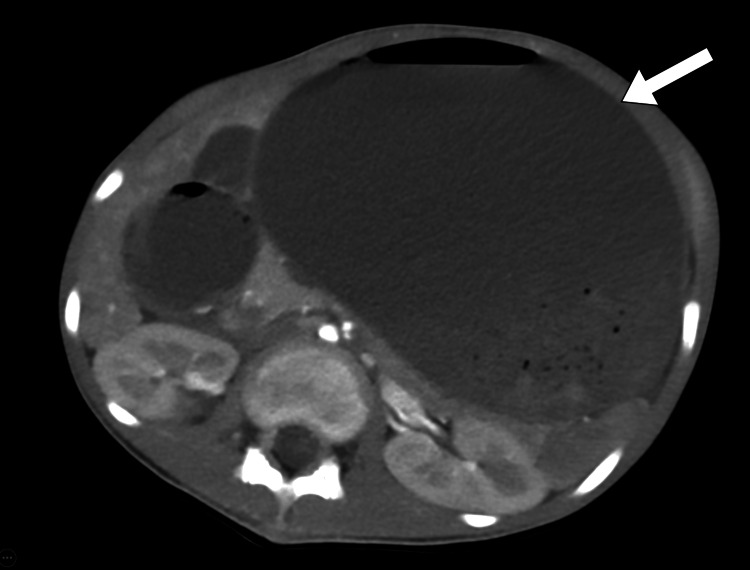
Axial CT scan of the abdomen shows a markedly fluid-filled stomach (arrow) causing a significant mass effect on the left kidney and adjacent bowel loops. CT: computed tomography

**Figure 3:**
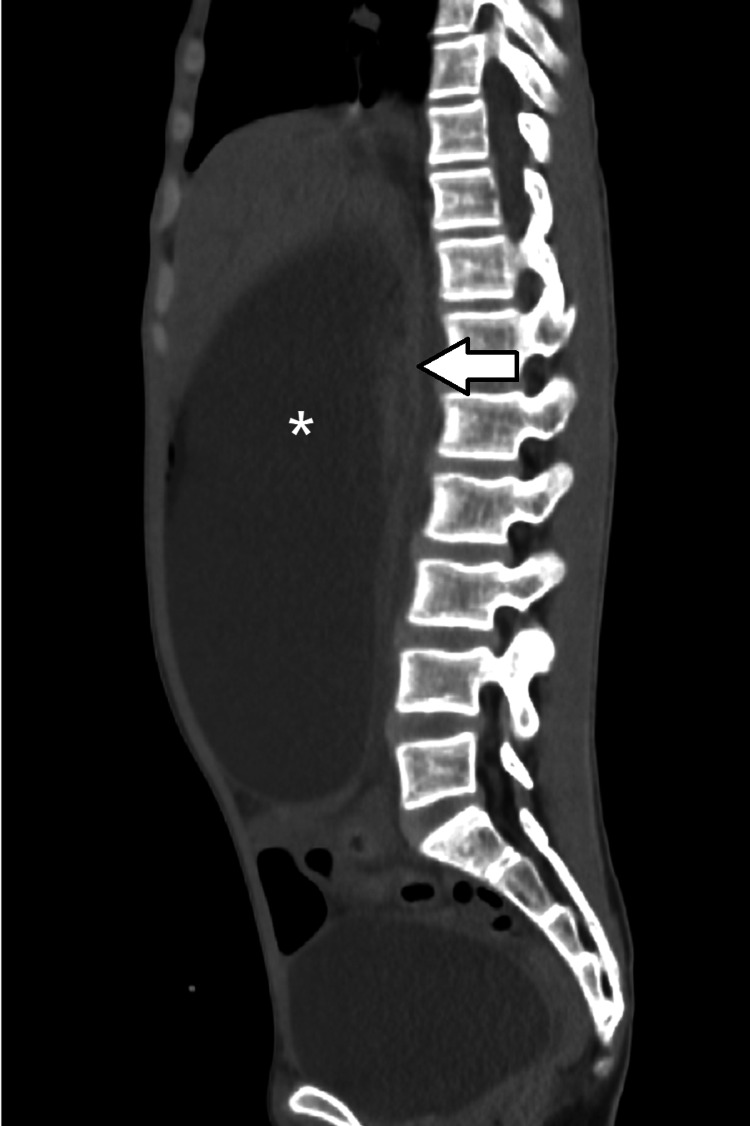
Sagittal CT scan of the abdominopelvic cavity shows a markedly distended stomach (asterisk) with a narrow aortomesenteric distance (arrow) consistent with superior mesenteric artery syndrome. CT: computed tomography

The plain abdominal radiograph showed a distended stomach with no abnormal small or large bowel dilatation (Figure 1). Computed tomography of the abdomen was performed to rule out intestinal obstruction. The scan demonstrated extreme dilation of the fluid-filled stomach and the proximal duodenum with a transition point in the aortomesenteric region. The stomach measures 20 cm x 14 x 18 cm on maximum dimensions (anteroposterior x mediolateral x craniocaudal). The distal part of the duodenum showed normal caliber. There was no pneumoperitoneum or ascites. The aortomesenteric angle measures 19 degrees (normal range: 28-65) and the aortomesenteric distance measured 8 mm (normal range: 10-34). A massively dilated stomach exerted a mass effect on adjacent structures and displaced the spleen and small intestinal loops inferiorly. The left renal vein was also compressed between the superior mesenteric artery and the aorta. The aforementioned findings were consistent with intestinal obstruction due to superior mesenteric artery syndrome together with nutcracker syndrome (Figures 2-3).

The patient had nasogastric tube insertion which drained 3.0 liters of bilious fluid. Aggressive intravenous fluid resuscitation was performed along with the correction of electrolyte disturbances. He received intravenous omeprazole 20 mg and ondansetron 8 mg as a symptomatic treatment for nausea and vomiting. A nasojejunal tube was inserted for enteral feeding. The patient showed a significant clinical improvement after five days, as he gained 0.8 kg. He was able to tolerate oral feeding and the nasojejunal tube was removed. The child protection team was involved in the patient’s management. The team concluded that the child received less care after the parents received genetic confirmation of the diagnosis of Down syndrome, leading to rapid weight loss and the development of superior mesenteric artery syndrome. The growth parameters record indicated that the patient lost 4 kg in two weeks since the diagnosis of Down syndrome was confirmed. The patient was discharged after extensive counseling with the parents together with a social worker. The child was seen in the clinic with regular visits and no active problems were observed.

## Discussion

The present case describes a case of proximal intestinal obstruction secondary to superior mesenteric artery syndrome in a patient with Down syndrome. Superior mesenteric artery syndrome is a rare condition in children. Biank et al. [3] conducted a retrospective review of medical charts over 20 years and identified only 22 cases of superior mesenteric artery syndrome in children.

Typically, superior mesenteric artery syndrome develops after a history of acute weight loss before the diagnosis [3]. In the present case, it is assumed that the child was neglected after the parents were confronted with the diagnosis of Down syndrome, which has led to rapid weight loss leading to the development of superior mesenteric artery syndrome. However, in any case, the history of weight loss is not reported. For example, an interesting report by Okamoto et al. [4] describes a case of superior mesenteric artery syndrome in a healthy adolescent with insufficient weight gain relative to height growth. In the pediatric population, superior mesenteric artery syndrome is typically seen in patients after corrective spine surgery for scoliosis [5], as this procedure lengthens the spine cranially and decreases the angle of the aortomesenteric.

A high index of suspicion for superior mesenteric artery syndrome is required given the non-specific clinical presentation. Imaging criteria for superior mesenteric artery syndrome involve duodenal obstruction with an aortomesenteric angle of less than 25 degrees and a high fixation of the duodenum by the ligament of Trietz [6]. The management of superior mesenteric artery syndrome is usually conservative. The goal of treatment is to decompress the bowel and correct any fluid and electrolyte disturbances. Surgical treatment is attempted when conservative therapy fails to achieve any improvement [6].

## Conclusions

Superior mesenteric artery syndrome is a rare cause of proximal intestinal obstruction in children. Weight loss is an important predisposing factor to this condition. Therefore, clinicians must maintain a high index of suspicion of superior mesenteric artery syndrome in any child who presents a clinical picture of intestinal obstruction after rapid weight loss for any reason. Conservative treatment with aggressive fluid resuscitation and correction of electrolyte disturbances is generally successful.
